# QT Is Longer in Drug-Free Patients with Schizophrenia Compared with Age-Matched Healthy Subjects

**DOI:** 10.1371/journal.pone.0098555

**Published:** 2014-06-02

**Authors:** Kumiko Fujii, Yuji Ozeki, Hiroaki Okayasu, Yumiko Takano, Takahiro Shinozaki, Hiroaki Hori, Masami Orui, Minoru Horie, Hiroshi Kunugi, Kazutaka Shimoda

**Affiliations:** 1 Department of Psychiatry, Dokkyo Medical University School of Medicine, Tochigi, Japan; 2 Department of Mental Disorder Research, National Institute of Neuroscience, National Center of Neurology and Psychiatry, Tokyo, Japan; 3 Department of Health Care, Dokkyo Medical University School of Medicine, Tochigi, Japan; 4 Department of Cardiovascular and Respiratory Medicine, Shiga University School of Medical Science, Shiga, Japan; Benito Menni Complejo Asistencial en Salud Mental, Spain

## Abstract

The potassium voltage-gated channel KCNH2 is a well-known gene in which mutations induce familial QT interval prolongation. KCNH2 is suggested to be a risk gene for schizophrenia. Additionally, the disturbance of autonomic control, which affects the QT interval, is known in schizophrenia. Therefore, we speculate that schizophrenic patients have characteristic features in terms of the QT interval in addition to the effect of antipsychotic medication. The QT interval of patients with schizophrenia not receiving antipsychotics (n = 85) was compared with that of patients with schizophrenia receiving relatively large doses of antipsychotics (n = 85) and healthy volunteers (n = 85). The QT interval was corrected using four methods (Bazett, Fridericia, Framingham or Hodges method). In ANCOVA with age and heart rate as covariates, patients not receiving antipsychotic treatment had longer QT intervals than did the healthy volunteers, but antipsychotics prolonged the QT interval regardless of the correction method used (P<0.01). Schizophrenic patients with and without medication had a significantly higher mean heart rate than did the healthy volunteers, with no obvious sex-related differences in the QT interval. The QT interval prolongation may be manifestation of a certain biological feature of schizophrenia.

## Introduction

The KCNH2 channel (which is also known by the name of herg), a member of the subfamily of voltage-gated K+ channels, is responsible for a delayed rectifier potassium current of myocardial cells, which is a major component of cardiac repolarization [1]. The KCNH2 channel gene variant causes a congenital form of long QT syndrome or predisposes an individual to acquiring long QT syndrome [2]–[3]. In addition, antipsychotics can induce QT interval prolongation [4]–[5]. The blockage of the KCNH2 channel via antipsychotics has been identified as a mechanism underlying QT interval prolongation from antipsychotic administration [6]. Therefore, a decline in KCNH2 channel function is assumed to be a mechanism of QT interval prolongation. In addition, several recent association studies have cited isoforms/polymorphisms of the KCNH2 gene as a risk factor for schizophrenia [6]–[9]. Moreover, the QT interval is affected by autonomic control [10], and studies of the autonomic system in schizophrenia have detected a number of abnormalities [11]. Therefore, we hypothesized that the prolongation of the QT interval in patients with schizophrenia might not only be caused by antipsychotic administration but also be characteristic of the disease.

Accordingly, to reveal the characteristics of QT intervals of schizophrenia excluding the effect of antipsychotic medications, we compared the QT interval of patients with schizophrenia not receiving antipsychotics with that of patients with schizophrenia receiving antipsychotics and healthy volunteers.

## Materials and Methods

### Subjects

Between 1996 and 2008, 111 drug-free schizophrenic patients were recruited at the National Institute of Neurology and Psychiatry Hospital, Japan. These participants were all non-adherent patients or naïve of antipsychotics. Whether a patient was drug free was judged based on the doctor's description in the medical record. According to medical records, no antipsychotic medication was prescribed to “drug-free” patients within several months before ECG recording. No patient was administered depot antipsychotics. Of the 111 patients, 23 with hypokalemia (K<3.5), two with a complete right bundle branch block, and one with a postoperative atrial septal defect were excluded from the study. No participant suffered from arrhythmia or other conduction disorders of the heart. Accordingly, data from 85 drug-free patients were analyzed. For the antipsychotic administration group, we selected 85 age-matched patients with schizophrenia (examined during the same time period as the first group) who were receiving pharmacotherapy and had no QT prolongation factors (e.g., hypokalemia, hypothyroidism, and/or ischemic heart disease) other than antipsychotic medication. To reveal the QT prolongation effect of antipsychotics more clearly, we sampled the patients who were administrated high-dose antipsychotics as the antipsychotic administration group [the mean chlorpromazine- equivalent dose of prescribed antipsychotic was 1799.7 mg (SD: 1454.5)]. Almost all of the patients were administered first-generation antipsychotics, and some of these medications have a relatively high anticholinergic effect (chlorpromazine and levomepromazine). First generation antipsychotics were administered to 76 patients [haloperidol: 53 patients [mean dose (SD) = 20.2 (12.2) mg], chlorpromazine: 32 patients [mean dose (SD) = 224.2 (208.0) mg]. levomepromazine: 31 patients [mean dose (SD) = 90.2 (68.5) mg]. On the other hand, only 3 patients were treated by monotherapy with a second generation antipsychotic (olanzapine 20 mg or risperidon 3 mg or 5 mg). The third group comprised 85 healthy volunteers who had undergone routine health check-ups between 2010 and 2012 at Dokkyo Medical University Hospital, Japan. These participants had no physical abnormalities or abnormal laboratory data.

Patients were diagnosed according to the DSM-IV-TR criteria. The demographic data for each group are shown in Table 1. One-way analysis of variance (ANOVA) revealed no significant differences in age or sex between the groups, although significant differences were observed for heart rate and smoking status. Namely, the groups were matched on age and the proportion of sex which are known to affect QT interval; however, the number of smokers with potential to affect the QT interval was not controlled.

**Table 1:** Demographic data of participants and comparison of QTc calculated with four types of formulae.

	Normal control	Schizophrenia (drug free)	Schizophrenia (medication)
N	85	85	85
Chlorpromazine equivalent daily dose (SD):mg/day	―	0	1799.7(1454.5)
Male (n, %)	38(45)	36(42)	40(47)
Age, years (SD)	42.5(5.6)	40.4(14.6)	40.2(14.0)
Smoking (n, %)*	19(22)	31(36)	33(39)
Pulse, beat per minute (SD)**	63.9(8.9)	81.0(19.8)	79.8(15.6)
QTcB(SD)^+^:msec	390.8(21.2)	406.1(25.0)	423.3(33.6)
QTcFri (SD)^+^:msec	387.2(18.4)	388.7(29.8)	405.3(36.2)
QTcFra (SD)^+^:msec	387.2(18.6)	390.5(27.1)	406.4(32.7)
QTcH (SD)^+^:msec	387.4(17.9)	394.6(25.4)	407.6(30.8)

Statistically Significant *p<.05; **p<.01 by one-way ANOVA

Statistically Significant ^+^p<.01 by ANCOVA.

This study is a retrospective cross-sectional study. The data we prepared were recorded for clinical use. All subjects for whom complete medical records were available were included in the study. From medical record, no participants were judged to use illicit substances. Based on physical examination and chest radiography, structural heart disease was considered unlikely in the included participants. We collected the data from clinical records after approval was given by the Institutional Review Boards of Dokkyo Medical University School of Medicine and the National Institute of Neurology and Psychiatry. The design of this study also follows the ethical norm from the Ministry of Health, Labor and Welfare of Japan.

In our approved method, written informed consent was not given by participants. To anonymize their clinical records which were used in this study, the information which can identify an individual (e.g. name and registration number) was not prepared. Furthermore, the clinical data were put together to one person and were managed. Another person who took charge of statistical analysis could not access original data of each individual. Because the all participated patients agreed with hospital treatment spontaneously and healthy controls underwent the examinations spontaneously, in the participants, there was no person who had a compromised capacity/ability to consent. The children were not included in the participants.

### Evaluation of the QT interval

The QT interval was measured manually according to previous report [12]. The end of the T-wave was determined as the intersection between the tangent to the steepest downslope of the T-wave and the isoelectric line. The QT interval was corrected as a value that varies in relation to heart rate. A number of methods for correcting the QT interval by heart rate have been proposed, each with distinctive characteristics. In the current study, we used four correction methods, as follows: methods from Bazett [13], Fridericia [14], Framingham [15], and Hodges [16], with the following correction formulae:

Bazett: 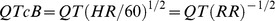


Fridericia: 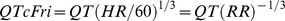


Framingham: 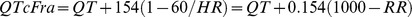


Hodges: 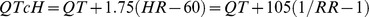 where HR is heart rate and RR is R-R interval.

### Datal analysis

Analysis of covariance (ANCOVA) was used to compare the QT interval corrected using the four correction methods described above (QTcB, QTcFri, QTcFra and QTcH) between drug-free patients with schizophrenia, medicated patients with schizophrenia and healthy controls. Different covariates were selected for the different comparisons and they are presented in the corresponding sections in the Results. Sex (male: 1, female: 2) and smoking status (smoking: 1, nonsmoking: 2) were also compared between the groups. Bonferroni's method for multiple comparisons was used to compare the simple main effect of the QT interval, and the level of significance was set at *P*<0.05. Statistical analysis was conducted using PASW statistics 18 (SPSS Inc., Chicago, IL).

## Results

Table 1 shows the demographic data and QT intervals corrected by the four methods for the drug-free patients, medicated patients, and controls.

The ANCOVA with age and heart rate as covariates showed a significant difference between the three groups regardless of the type of correction method used. Bonferroni's post hoc test revealed that the QT interval corrected using each of the four methods was significantly longer in medicated patients than in drug-free patients and controls. In addition, the corrected QT interval in drug-free patients was significantly longer compared to that of the controls (Table 2). These differences might be caused by differences in heart rate, as one-way ANOVA revealed a higher heart rate in the drug-free and medicated patients compared to normal controls and previous reports indicated that values corrected using the Bazett, Framingham and Fridericia formulae are more influenced by relatively higher heart rate than is the Hodges formula [17].

**Table 2:** Difference of main effects between each group.

	Difference of estimated marginal means (msec)			
	QTcB (SD)	QTcFri (SD)	QTcFra (SD)	QTcH (SD)
Schizophrenia(drug free)-normal control	19.6(5.3)**	20.1(5.0)**	19.8(4.6)**	21.5(4.5)**
Schizophrenia(medication)-normal control	38.5(5.2)**	37.5(4.9)**	36.5(4.5)**	35.4(4.4)**
Schizophrenia(medication)-schizophrenia(drug free)	18.9(4.3)**	17.4(4.0)**	16.7(3.7)**	13.8(3.6)**

Note, SD: standard deviation.

Statistically significant **p<.01.

Taking this into account, we focused on subjects with heart rate <90 beats/min to minimize the influence of heart rate on the results. The demographic data for each of the three groups are shown in Table 3. The data revealed a significant difference between the groups regardless of the type of correction method used (i.e., QTcB, QTcFri, QTcFra, and QTcH) when ANCOVA was performed with only age as a covariate. Bonferroni's post hoc test also revealed that QTcB, QTcFri, QTcFra and QTcH were significantly longer in medicated patients than in drug-free patients and controls. Additionally, QTcB, QTcFri, QTcFra and QTcH were significantly longer in drug-free patients than in the controls (Table 4).

**Table 3:** Demographic data of participants whose heart rate is less than 90 beats per minutes.

	Normal control	Schizophrenia (drug free)	Schizophrenia (medication)
N	83	55	65
Chlorpromazine equivalent daily dose (SD):mg/day	―	0	1654.2(1313.7)
Male(n, %)	37(45)	24(44)	31(48)
Age, years (SD)	42.4(5.5)	40.4(14.2)	40.3(14.3)
Smoking (n, %)*	19(45)	23(42)	28(43)
Pulse, beat per minute (SD)**	63.3(7.8)	68.9(11.9)	73.4(11.0)
QTcB (SD)^+^:msec	390.5(21.3)	408.8(25.2)	427.1(28.9)
QTcFri (SD)^+^:msec	387.5(18.5)	400.6(26.5)	414.0(29.8)
QTcFra (SD)^+^:msec	387.5(18.8)	401.4(24.7)	414.7(27.5)
QTcH (SD)^+^:msec	387.5(18.0)	401.4(26.1)	413.2(28.1)

Pulse<90.

Statistically Significant *p<.05; **p<.01 by one-way ANOVA

Statistically Significant ^+^p<.01 by ANCOVA.

**Table 4:** Difference of main effects between each group.

	Difference of estimated marginal means (msec)			
	QTcB (SD)	QTcFri (SD)	QTcFra (SD)	QTcH (SD)
Schizophrenia(drug free)-normal control	19.1(5.1)**	19.7(4.9)**	19.4(4.7)**	21.4(4.6)**
Schizophrenia(medication)-normal control	38.0(5.0)**	38.0(4.8)**	36.7(4.6)**	38.5(4.6)**
Schizophrenia(medication)-schizophrenia(drug free)	18.9(4.8)**	18.3(4.6)**	17.3(4.4)**	17.1(4.3)*

(heart rate of participants is less than 90 beats per minutes).

Note, SD: standard deviation.

Statistically significant *p<.05; ** p<.01.

The QT interval is known to differ between the sexes, with females having longer QT intervals than males. In support of this, we observed that female controls had longer QTcB, QTcFri, QTcFra and QTcH intervals than did male controls. However, importantly, no sex difference in terms of QTc interval was observed in the drug-free or medicated patients. We observed no sex differences in heart rate between the drug-free patients, medicated patients, or controls (Table 5).

**Table 5:** Comparison between gender in heart rate and QT interval by one-way ANOVA.

	Normal control	Schizophrenia (drug free)	Schizophrenia (medication)
**Heart rate(beats/min.)**			
Male	62.5(9.3)	81.1(21.5)	79.1(15.5)
Female	65.0(8.4)	80.8(18.6)	80.6(15.9)
**QTcB(msec)**			
Male	379.2(20.5)**	406.7(25.8)	424.7(36.6)
Female	400.2(16.6)	405.7(24.7)	409.1(26.2)
**QTcFri(msec)**			
Male	377.1(17.4)**	389.4(29.9)	391.6(31.5)
Female	395.3(15.0)	388.2(30.0)	395.5(28.9)
**QTcFra(msec)**			
Male	376.8(17.6)**	390.7(27.0)	392.0(29.3)
Female	395.7(14.9)	390.3(27.4)	396.9(26.7)
**QTcH(msec)**			
Male	377.9(17.4)**	396.2(27.6)	394.6(29.6)
Female	395.0(14.4)	393.4(23.9)	398.1(23.8)

Statistically significant **p<.01 by one-way ANOVA.

## Discussion

In this study, after adjustments for age and heart rate, patients with schizophrenia displayed longer QT intervals than healthy volunteers even when they were not receiving antipsychotics, although antipsychotics were shown to further prolong the QT interval. The magnitude of the difference in the QT interval between controls and drug-free schizophrenic patients is small. Furthermore, patients with schizophrenia had a higher heart rate than did the healthy volunteers. Whereas a sex difference was observed in the QT interval in the normal controls, no such difference was found in the patients with schizophrenia. In schizophrenia, studies of the autonomic system, which affects the QT interval [10], have detected a number of abnormalities. For example, decreased heat rate variability, a marker of cardiac parasympathetic activity has been demonstrated in patients with psychosis [18]–[20]. Furthermore, increased variability in the QT interval has been reported in first episode-neuroleptic naïve psychosis [11] as well as in schizophrenic patients and their relatives [19], suggesting that schizophrenia itself is the risk factor for QT interval abnormality.

The KCNH2 channel is thought to be involved in the molecular biological mechanism of pre-existing prolonged QT intervals in schizophrenic patients for several reasons. First, the KCNH2 channel is expressed in myocardial cells and is related to the early components of the inward current necessary for repolarizing myocardial cells [1]. Second, some patients with familial long QT syndrome suffer from genetic KCNH2 abnormalities [2]–[3]. Third, blockage of the KCNH2 channel is a probable molecular mechanism for antipsychotic-induced QT interval prolongation [21]. Fourth, association studies have shown that the KCNH2 channel is associated with schizophrenia [6]–[9]. It was also recently discovered that KCNH2 channels are expressed in the brain, including the midbrain dopamine neurons, where they are expected to alter dopamine release [21].

Another genetic factor that is considered to be involved in pre-existing QT interval prolongation in schizophrenic patients is the neuregulin 1 gene (NRG1), which Stefansson et al. reported as potentially related to the onset of schizophrenia [22]. NRG1 plays various roles in the central nervous system, promotes the differentiation of myocardial cells of the cardiac conduction system [23], and is thought to be related to the control of cardiac autonomic nervous balance [24]. Therefore, NRG1 could be a candidate factor for explaining the prolongation of the QT interval observed in the patients with schizophrenia in the present study. In addition, a report recently indicated that a common missense variant in the NRG1 gene is associated with both schizophrenia and sudden cardiac death [25]. This report might indicate the association between NRG1 and QT interval prolongation, as QT interval prolongation is the most widely used surrogate marker for assessing the risk of torsades de pointes (TdP), one of the causes of sudden cardiac death, although the QT interval is considered somewhat imprecise as a surrogate marker for TdP [26]. Furthermore, the present finding that the heart rate of patients with schizophrenia was increased compared to controls is consistent with previous reports [19], [27] and with the finding that the heart rate of mice with impaired NRG1 function was higher than that of control animals [28].

Healthy female volunteers have significantly longer QT intervals than healthy males [13]. However, some studies on QT intervals in patients with schizophrenia receiving antipsychotics have reported no significant sex difference [5], [29]–[30]. For example, Ramos-Rios et al. reported that the mean corrected QT (QTc) for male schizophrenic patients aged >50 years exceeded that for female patients of the same age, whereas no significant difference was found between the mean QTc of male and female patients aged <50 years [31]. The present study found a sex difference in the QTc interval of healthy controls but not in patients with schizophrenia. Although sex differences in the symptoms and prognosis of schizophrenia have been reported [32], it is conceivable that the influence of factors that are involved in the etiology of schizophrenia (e.g., KCNH2 or NRG1) on the QT interval might be sufficient to negate the sex difference observed in healthy controls.

In the present study, four types of QT interval correction formulae were applied, although the Bazett formula seems to give higher QTc values in patients with schizophrenia but not in controls, compared to the other corrections. The Bazett correction is widely used in clinical practice but may thus not be the best choice in schizophrenia.

This study has some limitations. The number of participants is relatively small because of the difficulty in preparing the ECG data of drug-free schizophrenic patients. Each participant underwent a single ECG examination. In addition, the measurement timing was not fixed, although it has some diurnal variation. Obesity and binge drinking have been indicated as factors affecting the QT interval [33]–[34], but we did not estimate such factors in this study. Structural heart disease, which is one of the most important variables responsible for QT prolongation, could not be excluded completely because echocardiography was not performed. However, all participants underwent physical examination and chest radiographic inspection. Therefore, we decided that in participants, the probability of suffering from structural heart disease was very low and should hardly affect the results.

In conclusion, this study found that patients with schizophrenia, even when not receiving antipsychotics, had a longer QT interval than healthy volunteers. Additionally, the QT interval was further prolonged by the administration of antipsychotics. Although the QTc interval is considered somewhat imprecise as a surrogate marker for assessing the risk of TdP, we speculate that the QT interval may be manifestation of a certain biological feature of schizophrenia.
